# The Sensitivity of the Crayfish Reward System to Mammalian Drugs of Abuse

**DOI:** 10.3389/fphys.2017.01007

**Published:** 2017-12-06

**Authors:** Adam T. Shipley, Adebobola Imeh-Nathaniel, Vasiliki B. Orfanakos, Leah N. Wormack, Robert Huber, Thomas I. Nathaniel

**Affiliations:** ^1^Department of Biomedical Sciences, University of South Carolina School of Medicine, Greenville, SC, United States; ^2^Department of Biology, North Greenville University, Tigerville, SC, United States; ^3^J.P Scott Center for Neuroscience, Mind and Behavior, Bowling Green State University, Bowling Green, OH, United States

**Keywords:** amphetamine, appetitive motor patterns, crayfish, drugs of abuse, exploratory behavior

## Abstract

The idea that addiction occurs when the brain is not able to differentiate whether specific reward circuits were triggered by adaptive natural rewards or falsely activated by addictive drugs exist in several models of drug addiction. The suitability of crayfish (*Orconectes rusticus*) for drug addiction research arises from developmental variation of growth, life span, reproduction, behavior and some quantitative traits, especially among isogenic mates reared in the same environment. This broad spectrum of traits makes it easier to analyze the effect of mammalian drugs of abuse in shaping behavioral phenotype. Moreover, the broad behavioral repertoire allows the investigation of self-reinforcing circuitries involving appetitive and exploratory motor behavior, while the step-wise alteration of the phenotype by metamorphosis allows accurate longitudinal analysis of different behavioral states. This paper reviews a series of recent experimental findings that evidence the suitability of crayfish as an invertebrate model system for the study of drug addiction. Results from these studies reveal that unconditioned exposure to mammalian drugs of abuse produces a variety of stereotyped behaviors. Moreover, if presented in the context of novelty, drugs directly stimulate exploration and appetitive motor patterns along with molecular processes for drug conditioned reward. Findings from these studies indicate the existence of drug sensitive circuitry in crayfish that facilitates exploratory behavior and appetitive motor patterns via increased incentive salience of environmental stimuli or by increasing exploratory motor patterns. This work demonstrates the potential of crayfish as a model system for research into the neural mechanisms of addiction, by contributing an evolutionary, comparative context to our understanding of natural reward as an important life-sustaining process.

## Introduction

As individuals experience repeated exposure to opiates and other psychoactive drugs, vulnerable individuals enter an addictive cycle that is triggered by several mechanisms. These drugs initially function as reinforcers that strengthen behaviors associated with drug intake. After a short period of time, the resulting tolerance and dependence lead to progressively higher doses to maintain a desired effect (Wise and Koob, 2014). At this stage, compulsive drug-seeking behaviors become evident, even when paired with negative consequences (Wise, 1998). To identify useful targets for the development of future therapeutic interventions for drug-seeking behaviors, several studies explored the central components of drug-sensitive reward processes in both vertebrate and invertebrate species. Much of these efforts have been focused on an evolutionary basis of drug reward as an entrenched process within natural reward systems (Higgins and Fletcher, 2003; Panksepp and Huber, 2004; Nathaniel et al., 2009; Huber et al., 2011). Findings from most of these studies reveal that mammalian drugs of abuse typically exploit the natural reward systems, which align with the species' adaptive needs. These drugs function by supplanting the individual's inherent pursuit of its basic needs, such as nourishment, shelter, and reproduction, with a search for the drug instead (Koob and Le Moal, 2001). Findings from these studies provided the opportunity to investigate common neural substrates underlying reward in a model system that has previously shown remarkable success under similar conditions and, to date, has provided major insights into wide-ranging behavioral occurrences. The first part of this review discusses the core neural pathways associated with drug addiction. The importance of invertebrate model systems in drug addiction research is then highlighted. Finally, a series of experiments that support crayfish as a powerful invertebrate model system for the study of drug addiction are discussed.

## Neural pathways in drug addiction

Dopamine is considered the primary neural pathway underlying the neural causations of excitement, curiosity, and exploration (Alcaro et al., 2007). Several studies in the past have challenged a unitary role of the pathway in “pleasure.” The common neural pathways surrounding mesolimbic dopaminergic neurons are commonly thought to mediate subjective reward and maintain reinforcement processes via hedonic affect (Schultz, 1997). Dopamine alters behavior via incentive salience in which motivational components are applied to stimuli that have shown to be rewarding in the past (Johanson et al., 1976; Robinson and Berridge, 1993; Spanagel and Weiss, 1999). Mesolimbic and neostriatal dopamine systems exhibit residual reward capacity even after depletion of dopamine, which demonstrates a value in learning that is independent of hedonia and strict reward-based learning (Berridge and Robinson, 1998). The concept of “wanting” has been defined from the idea of reward-related stimuli conferring a motivational value to an organism, which is distinct from hedonia (Panskepp, 1998; Panksepp, 2005). The “wanting” mechanism may be modulated by dopamine systems via perceived attractiveness, rather than the traditional view of receiving pleasure, or “liking” a stimulus.

The distinction between “wanting” and “liking” is important as it appears that drug-mediated dopamine responses progress by “wanting” something more but “liking” it less (Robinson and Berridge, 2001; Berridge and Robinson, 2003). Drugs can be associated with certain contextual cues, such as a novel environment. For example, when an organism is conditioned to receive a psychoactive drug paired with a sensory cue, associated neural functions are activated in response to the environmental cue. In the absence of the drug itself, the effect goes so far to re-activate and sustain drug seeking behavior (Davis and Smith, 1976; Cervo et al., 2003; Burbassi and Cervo, 2008).

The dopaminergic pathways are responsible for feelings of desire and reward in humans through their influence on the ventral tegmental region, medial forebrain bundle and the nucleus accumbens (Alcaro et al., 2007), and can modulate compulsive behavior characteristic of drug addiction in several mammalian models. Dopamine is also implicated in a more direct learning process, in which mesolimbic dopamine neurons fire unconditionally in affiliation with natural rewards often associated with survival. Over time, however, this dopaminergic activity will shift from firing in response to the reward itself to firing in response to the cue that is predictive of the novel reward (Schultz, 1997; Vanderschuren and Kalivas, 2000). Although reward can be grouped into a few separate processes; an object's incentive value, the connective learning process of predictive cues and the object of attraction including the object's ability to produce hedonism are distinct in their own way and they each relate to a dopaminergic response that reinforces reward (Wise, 1998; Ikemoto and Panksepp, 1999; Kelley, 1999; Everitt et al., 2001; Panksepp and Huber, 2004). It is seemingly paradoxical, that humans and animals are susceptible to addictive effects of cocaine, a neurotoxic chemical that has been shown to be evolutionarily adapted to protect the coca plant from insect herbivory by interfering with motor control in the organisms that consume coca plant (Nathanson et al., 1993). The dopaminergic system should be affected by cues that provide reward, not a plant neurotoxin that is designed to thwart predation. Several theories have been proposed that attempt to provide an evolutionary explanation for this phenomenon, ranging from co-evolution of herbivores and plants, to simple fundamental differences in response to the chemical by mammals compared to arthropods (Nathanson et al., 1993).

## Invertebrate model systems in drug addiction research

The introduction of invertebrate model systems in evolutionarily relevant studies of drug-induced reinforcement, compulsion, withdrawal, reinstatement, and addiction has greatly broadened this field of research. These systems have shown to be powerful tools in the understanding of the neuroanatomical and behavioral processes underlying the addictive process. Benefits of invertebrates, aside from being more cost effective, offering reduced moral concerns, and behaviors patterned by experimentally accessible neural structures, are shared homologies with mammals in the key neurochemical aspects of reward, including receptor elements (Hen, 1992a, 1993), neuropharmacology (Tierney, 2001), mechanisms of action (Vernier et al., 1995, 1997), deactivation (Pörzgen et al., 2001), and association with similar behavioral contexts (Kravitz et al., 1980; Kravitz, 2000). Monoamine systems developed during the transition to metazoan life, where they were used to adapt functions of individual cells to disturbances within their environment (Gillette, 2006). Importantly, dopamine and serotonin receptors predate the chordate lineage (Hen, 1992b; Peroutka and Howell, 1994; Vernier et al., 1995; Walker et al., 1996), and divergence has given rise to considerable diversity in specific subtypes within different lineages, along with some unique differences in receptor subunits and pharmacological properties in both vertebrates and invertebrates. As a result of the divergence during evolution, mammals utilize oxidation and methylation while flies use N-acetylation and β-alanylation for dopamine (DA) metabolism (Yamamoto and Seto, 2014). Indeed, flies lack the genes required to synthesize norepinephrine and epinephrine, and these are two major catecholamines derived from DA that function in neuromodulation signaling in mammals (Yamamoto and Seto, 2014). A cloned dopamine receptor from *D. melanogaster* has similar structural and functional properties with vertebrate D1-type receptors, but the pharmacological properties are very different (Gotzes and Baumann, 1996; Schetz et al., 2011). The characterization of the sensitivity of *D. melanogaster* to cocaine in an *in situ* hybridization study demonstrates that dopamine transporter (dDAT) lacks all the structural components that are found in the mammalian catecholamine transporters (Pörzgen et al., 2001). Moreover, cocaine displayed a lower affinity for dDAT when compared with serotonin transporter (Pörzgen et al., 2001). This study provides evidence that the structural and pharmacological profiles of dDAT is different from the DAT of vertebrate species. In addition, it indicates that injected cocaine, methamphetamine or morphine agonists or antagonists may function differently in vertebrate and invertebrate models of addiction. Despite the differences that exist between vertebrates and invertebrates, crayfish, *D. melanonogaster* and other invertebrate model systems will continue to provide new insights into the regulatory mechanisms of DA signaling drug addiction research.

With the expansion of drug-addiction research into invertebrate models, identification of behavioral stereotypes and profiles have become evident (Palladini et al., 1996; McClung and Hirsh, 1998; Torres and Horowitz, 1998). Fruit flies are a popular model system and have been shown to behaviorally sensitize in a fashion similar to that of the mammalian neurochemical and behavioral response to psychostimulants (Pierce and Kalivas, 1997; Berridge and Robinson, 1998; Ikemoto and Panksepp, 1999). Behavioral sensitization in fruit flies is regarded to have an opposite effect of tolerance and is characterized by an increased intensity of drug cravings and associated behaviors (Robinson and Berridge, 1993). Strengthening the argument for invertebrate models, an important commonality between the two models suggests that catecholamine circuits in flies bear a strong resemblance to the mammalian sensitization process (Wolf, 1999; Wolf and Heberlein, 2003). For behavioral sensitization to occur in both flies (Li et al., 2000) and rats (Kalivas, 1995), stimulation of the pre-synaptic monoamine sites must occur. The post-synaptic sites also play an important role in the cocaine response as flies that under-express these receptors exhibit a reduced response to an initial exposure to the drug (Li et al., 2000). The opposite is true for mutants that over-express the receptor. In each of these mutant cases, the flies will not sensitize as the wild-type flies do. Vertebrate dopamine receptor antagonists can block cocaine-induced behaviors in fruit flies (Torres and Horowitz, 1998) and planarians (Palladini et al., 1996), strongly suggesting that dopamine is implicated in the resulting altered motor behaviors. Tyramine has been revealed as a vital part of the sensitization process in a number of animal models, including drosophila. Mutant individuals exhibiting lowered amounts of this amine are affected normally by the initial effects of cocaine but are less likely to sensitize. An increase in the individual's tyramine will result in a stereotypical sensitization akin to the wildtype counterparts (McClung and Hirsh, 1999). The *per* gene has an interactive role with tyramine, in that those lacking the gene will not undergo a normal sensitization process when stimulated with a vertebrate D2 agonist (Andretic et al., 1999; Andretic and Hirsh, 2000). The recent work revealing the activity of tyramine and the *per* gene in invertebrates has suggested that these processes could be conserved across a wide range of taxa. Tyramine has been likened to amphetamine's pharmacological profile as it inhibits membrane transporter uptake and alters synaptic catecholamines (Sitte et al., 1998). This work on the transcription of the *per* gene has led to its demonstration in mammalian dorsal striatal regions receiving input from midbrain dopaminergic neurons (Nikaido et al., 2001). A recent study (Northcutt et al., 2016) identified genes for 34 distinct ion channel types, 17 biogenic amine and 5 GABA receptors, 28 major transmitter receptor subtypes including glutamate and acetylcholine receptors and 6 gap junction proteins—the innexins in the nervous system of Jonah crab (*Cancer borealis*) and the American lobster (*Homarus americanus*). These genes are associated with neural function in the crustacean systems and could provide important new insights to understand the organization of circuits in the control of behaviors. Other recent studies (Søvik et al., 2014; Zhu et al., 2014; Davies et al., 2015; Grotewiel and Bettinger, 2015; Hawkins et al., 2015; Engleman et al., 2016) indicate that an invertebrate system is a powerful tool that can be used to investigate the neuroanatomical, molecular and behavioral processes underlying the addictive process. Highlighting these accomplishments is vital in showing how simpler model systems can lead to exploration and discovery in mammalian systems as well.

The desire to more firmly establish invertebrate models in the study of drug addiction is driven by the lower cost and easy genetic manipulability of invertebrate models. To prove their effectiveness as a model, the biological and behavioral overlap between the two separate model systems needs to be demonstrated. The invertebrate model has been well established in the rewarding properties for psychostimulants (Wolf, 1999; Kusayama and Watanabe, 2000; Panksepp and Huber, 2004; Müller et al., 2007), opioids (Vanderschuren et al., 1997; Srivastava and Singh, 2006; Nathaniel et al., 2009, 2010), alcohol (Parsons, 1980; Bellen, 1998; Cadieu et al., 1999; Abramson et al., 2000, 2004), nicotine (Singaravelan et al., 2005), and caffeine (Singaravelan et al., 2005). Analogous to mammalian models, invertebrates also exhibit behavioral and motor stereotypes after the administration of cocaine. These studies show that fruit flies (McClung and Hirsh, 1998; Torres and Horowitz, 1998) and planarians (Palladini et al., 1996) exhibit increased locomotion and appetitive activities (Bellen, 1998; Torres and Horowitz, 1998; Wolf, 1999; Kusayama and Watanabe, 2000) which strongly resemble corresponding behaviors in mammals. Fruit flies have also been shown to demonstrate functional tolerance via a central nervous system adaptation with the administration of ethanol, mimicking mammalian tolerance and behavioral adaptation (Scholz et al., 2000). Land snails learn to self-administer electric current pulses into areas of the brain associated with sexual behavior (Balaban and Chase, 1991) and not administer treatments for areas controlling escape. This suggests that land snails feature distinct pathways involved with reward and punishment (Balaban, 1993; Balaban and Maksimova, 1993). Planarians exhibit susceptibility to place conditioning, as individuals will switch to non-preferred environments if it is paired with a psychostimulant. This effect could be subsequently blocked by administering selective vertebrate D1 and D2 antagonists (Kusayama and Watanabe, 2000).

## Crayfish as an invertebrate model of drug addiction research

Some crayfish-specific benefits in drug addiction studies includes a body size that allows for easy handling and a relatively simple neuroanatomical composition. Moreover, the crayfish amine system consists of fewer than 1,000 neurons, including 30–35 dopamine neurons in the brain and nerve cord (Furshpan and Potter, 1959; Tierney, 2001), axons with far reaching projections and large somata (Tierney et al., 1999), and a complex and easily identifiable behavioral set that offers convenient experimentation. Crayfish have already proven their effectiveness in exploring the proximate neural mechanism of behavioral decisions (Mulloney, 2003) and neurochemical mechanisms in neuroethological studies (Panksepp and Huber, 2002), showing their diverse uses outside of modeling reward to psychostimulants. The extensive usage of crayfish and lobster in various neuroethological studies (Livingstone et al., 1981; Edwards et al., 2003) has led to their use in studies for drug reward. The neuroanatomical and physiological characteristics of the crayfish allow for easy accessibility in pharmaco-behavioral manipulative studies (Huber and Delago, 1998; Panksepp and Huber, 2002), and evidence for conserved monoamine re-uptake systems in invertebrates (Corey et al., 1994; Demchyshyn et al., 1994; Pörzgen et al., 2001) showcase their ability to demonstrate mechanisms of reward resulting from psychostimulant administration (Robinson and Becker, 1986).

An initial set of experiments (Panksepp and Huber, 2004) characterized behavioral and locomotor effects for intracardial infusions of cocaine and amphetamines. For example, introduction of cocaine produced rapid backwards walking, waving of the claws, and escape behavior, such as tail flips. “Static posturing” was exhibited where the crayfish flexes the abdomen and walking legs, with claws pointed anteriorly and downward. Amphetamines induced muscle tremors in the walking legs, as well as the crayfish moving to the corner of the aquarium and appearing to investigate the surrounding with its antennae. A subsequent study of morphine injections resulted in an overall increase of exploration of the environment with recognizable patterns of locomotion and antenna movements (Nathaniel et al., 2010). Stimulated by tactile and olfactory cues to the antennae and antennules, this information is processed by the olfactory lobes and modulated by serotonin and dopamine (McMahon et al., 2005; Sullivan and Beltz, 2005; Patullo and Macmillan, 2006). Moreover, this site is recognized for its role in the rewarding action of cocaine and other psychostimulant addictive drugs (Nathaniel et al., 2012b).

## Drugs of abuse augment stereotypic behaviors (unconditioned studies)

**Figure 1:**
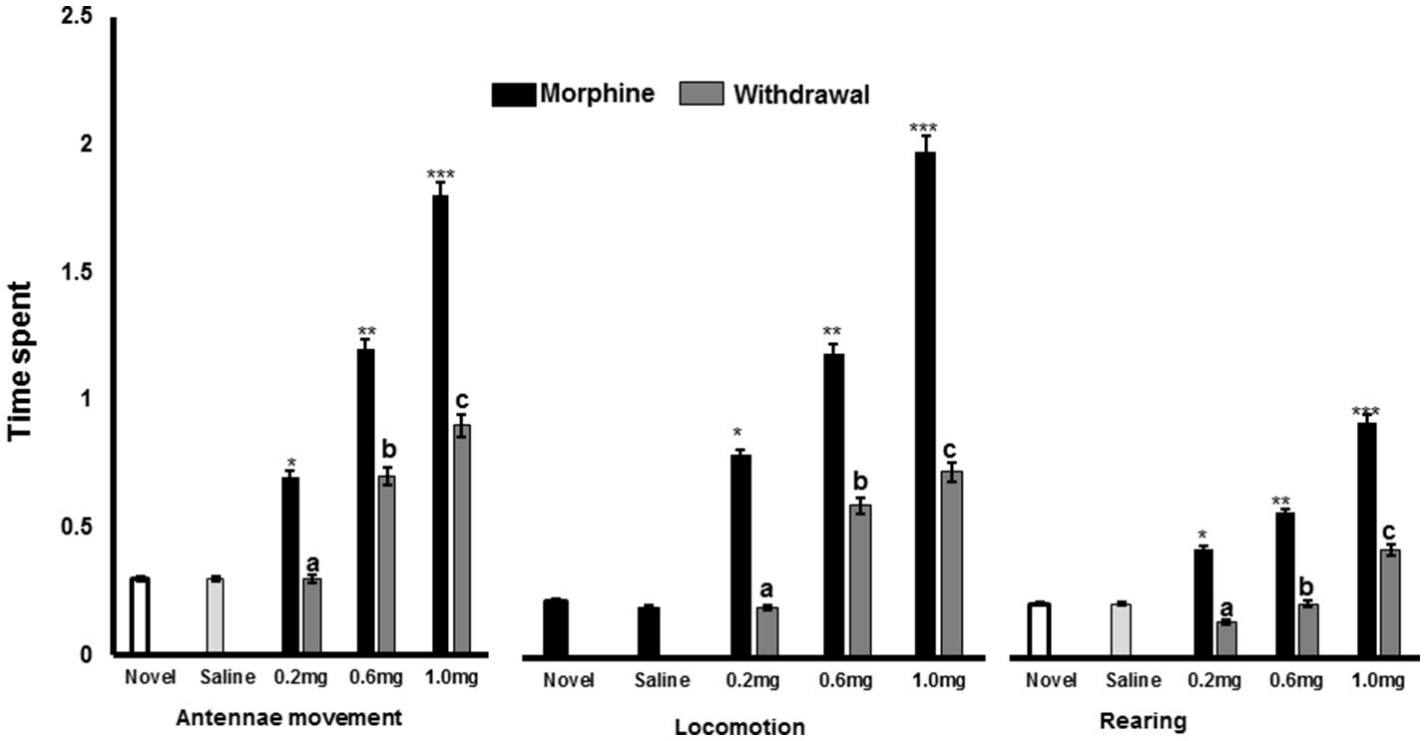
Effects of repeated morphine injections for 5 days on crayfish exploratory behaviors. Results are presented as mean percentage of time. *Post hoc* analysis revealed differences for 0.2 mg/kg (^a^*P* < 0.01), 0.6 mg/kg (^b^*P* < 0.001), and 1.0 mg/kg (^c^*P* < 0.0001) for antennae movements, locomotion, and rearing behaviors. Morphine injections increased antennae movements, locomotion and rearing behaviors for 0.2 mg/kg (^*^P < 0.01), 0.6 mg/kg (^**^P < 0.001), and 1.0 mg/kg (^***^P < 0.0001) doses.

Exploration is a major component of the reward system that exists in the crayfish model of drug addiction. An expression of appetitive motivational states, exploration entails various approaches in seeking a reward or positive outcome. Mammalian drugs of abuse promote unconditioned behavioral responses along with increased exploratory activity through approach behaviors (Panksepp and Huber, 2004). Approach behaviors such as the use of tactile and visual information are displayed in everyday life when searching for natural rewards such as food and shelter. In other words, mammalian drugs of abuse are particularly powerful in their ability to gain control of exploration behaviors, as the brain cannot distinguish whether reward circuits are being activated by genuine natural reward stimulus (such as food and shelter) or are being falsely triggered by psychostimulants, particularly amphetamine, cocaine, and morphine (Nathaniel et al., 2012b). When injected with drugs, the neural processes involved in appetitive motivation are stimulated and exploratory behavior is enhanced. The specific and differential effects of psychostimulant drugs (cocaine, amphetamine), and opioids (morphine) on the unconditioned behavioral response of crayfish at different doses over a period of 3 days was investigated (Nathaniel et al., 2012b). There was a significant effect of drugs on mobility when compared to the control group irrespective of drug. In a conditioning testing, morphine significantly increased locomotion at different doses (0.2, 0.6, and 1.0 mg), while locomotion was reduced in crayfish following repeated saline injections or withdrawal for 5 days in the previously morphine paired gravel background arena (Figure 1). This result indicates that paring with saline in the absence of morphine provided measures of the incentive properties of morphine in crayfish. For this reason, the reduction in exploratory behavior in the absence of response contingent drug availability probably led to the observed decline in the significance of the drug-paired stimuli in crayfish. Exploration of the environment as shown by patterns for locomotion, rearing and antenna movements increased in crayfish that were tested in the gravel environment, compared to crayfish that were tested in the plain background environment. The results indicate that novel stimuli can directly promote exploratory behaviors that are typically used to search for natural rewards.

In a qualitative analysis of drug-induced stereotypic behavior in crayfish, all three drugs produced distinct stereotypic behaviors. Following the cocaine injections at both low and high doses, crayfish adopted a static posture with legs flexed below the thorax and claws held downward. They remained static only for a few minutes before becoming mobile again. Following the low dose amphetamine injection, crayfish slowly approached the corners of the aquarium and consistently contacted the walls with their antennae. There was no static posture following the high dose amphetamine injection, but walking leg tremors, grooming, and perimeter exploration were observed. Low dose of cocaine injections produced enhanced rearing, antennae movement, and exploration of the corner of the experimental aquarium (Imeh-Nathaniel et al., 2017). A prior study investigated the effect of cocaine on specific locomotive traits (Nathaniel et al., 2012a), where intrapericardial injections of repeated doses of cocaine over the course of 3 days, decreased dose dependent lingering, increased speed of locomotion, distance traveled, and mobility, as well as increased immobility. This result suggests that each sub-component of locomotion is targeted by the effects of cocaine. The increased immobility is attributed to a potential desensitization of the involved receptors. These results revealed cocaine can produce distinct effects on movement and non-movement activities, indicating that cocaine impacts crayfish behavior in a way that is more specific to sub-locomotion components facilitated by the injected drugs.

Since repeated injections of cocaine are known to alter patterns of locomotion in crayfish, other studies in crayfish determined the relationship of single and repeated morphine injections on immediate and long-term effects of unconditioned behavior in crayfish. Significant effects of dose and time for single and repeated morphine treatments compared to saline controls, produced comparable long-term effects on locomotion. Even 5 days post treatment, these effects were maintained. These findings suggest that single and repeated doses of morphine can induce long-term behavioral sensitization including grooming, tail-flipping, movement of mouthparts, continuous exploration of aquarium corners, and mild tremors in the walking legs (Nathaniel et al., 2009).

## Novel stimuli directly augment exploration and appetitive motor patterns in crayfish (conditioned studies)

Drug addiction studies in humans, mammals, and more recently, crustaceans, utilize conditioned place preference (CPP) paradigms to examine the rewarding effects of mammalian drugs of abuse. CPP illustrates that a psychostimulant paired with an environmental cue increases preference for the latter, with dopamine neuronal activity shifting from direct association with the stimulant to the presentation of the environmental cue (Waelti et al., 2001). In such instances, even in the absence of the drug, the conditioned cue is sufficient to re-establish drug seeking behaviors in an individual (Davis and Smith, 1976). In crayfish a CPP protocol was used to examine unconditioned preferences for environments, followed by a drug-paired, conditioning phase and CPP test.

**Figure 2:**
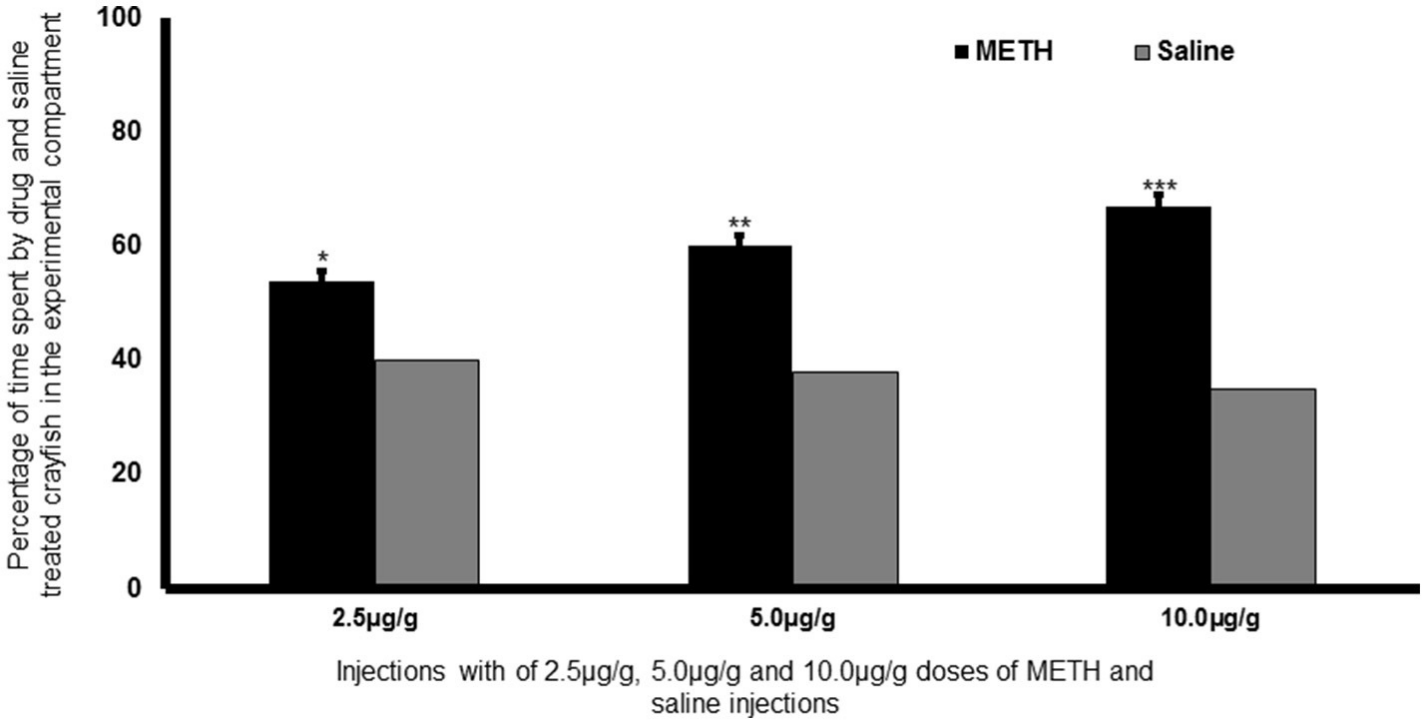
Repeated infusions of METH induced CPP in crayfish in the hard-textured experimental arena. There was a significant preference for the hard-textured compartment vs. soft textured-compartment following 5 days of injections with 2.5, 5.0, and 10.0 μg/g doses of METH, such that a conditioned place preference was established. *Post hoc* test comparison indicates that crayfish treated with 5.0 and 10.0 μg/g (^***^P < 0.05 and ^**^P < 0.01) were higher and different from the crayfish treated with 2.5 μg/g of METH (^*^P < 0.001) when compared with the saline-paired crayfish. The METH-conditioning effects were shown when crayfish that were treated with METH were paired with the naturally non-preferred hard environment. ANOVA [*F*_(4, 30)_ = 21.13; *P* < 0.001] reveals a significant effect, indicating a larger amount of time being spent in the METH-paired, hard-textured compartment when compared to saline conditioning, such that a conditioned place preference was established. The ANOVA factor revealed that the METH conditioning effect on crayfish was high (statistical power; 1 - β = 1.00) indicating that METH-induced CPP can be consistently replicated with a high degree of reliability.

In three different doses (2.5, 5.0, and 10.0 μg/g), methamphetamine induced a significant CPP for the hard-textured environment (Figure 2), with the higher doses (5.0 and 10.0 μg/g) of both drugs having a more pronounced effect of CPP. CPP was not established in the initially preferred soft textured environment when compared with the control group (Imeh-Nathaniel et al., 2016). Similarly, in a study investigating the effects of different visual cues on CPP when paired with morphine, crayfish initially showed an unconditioned preference for a white walled environment (Dziopa et al., 2011). After conditioning, crayfish showed preference for striped environment when paired with single and multiple morphine injections, at all doses.

These results were consistent with a previous study when crayfish was paired with environment showing textural differences (Nathaniel et al., 2009). The similarity in findings from these studies indicate that irrespective of the drug or its dosage, mammalian drugs of abuse prove to be rewarding to the crayfish when paired with a textural or visual environment. The significance of this observation is that the textural and visual stimuli are novel to the crayfish. An important question relevant to this review is, “how do crayfish find the hard texture novel?” As part of adaptation, the crayfish's brain is able to integrate appetitive motor responses such as seeking out for food and shelter. Their preference for a hard environment may be related to the intrinsic capability to use tactile cues, such as in the test environment, for survival. It is possible that crayfish might have explored and perceived the hard texture to be relatively attractive or novel when compared with the soft environments, suggesting that stimulus salience when paired with drugs indicates the significance or noticeability of the hard texture or striped visual environment as novel by crayfish. Similar findings were shown when varying doses of amphetamine were injected into the crayfish head ganglion during exposure to a novel environment (Alcaro et al., 2011). The administration of psychostimulants directly into the head ganglion enhanced active exploration of the novel environment. This indicates that the dopamine-mediated appetitive motivational states stimulated by drugs of addiction, conditions animals to pursue objects and environments for survival. It is possible that such an effect may enhance an adaptive behavior including exploration, and the acquired affective incentive value for cues associated with natural and drug rewards (Imeh-Nathaniel et al., 2016).

Exploratory behaviors such as locomotion, rearing, and antennae movements enhanced the ability of crayfish to seek rewards. A previous study characterized morphine-induced conditioned exploratory patterns and quantified atypical behaviors associated with termination of drug administration (Imeh-Nathaniel et al., 2014). In this study, when morphine was paired with a novel environment, locomotion, antennae movements, and rearing were enhanced in crayfish. Changes in exploratory behavior were diminished when morphine treatments were terminated and saline injections were given instead for five days. Locomotion was still elevated in withdrawal animals when compared to the saline control suggesting that morphine priming can reinstate an already established increase in locomotion irrespective of dose. This observation reveals the effects of morphine induced locomotion as well as the ability to restore exploratory behavior after extinction (Imeh-Nathaniel et al., 2014).

## Molecular alterations associated with drug conditioned rewards in crayfish

**Figure 3:**
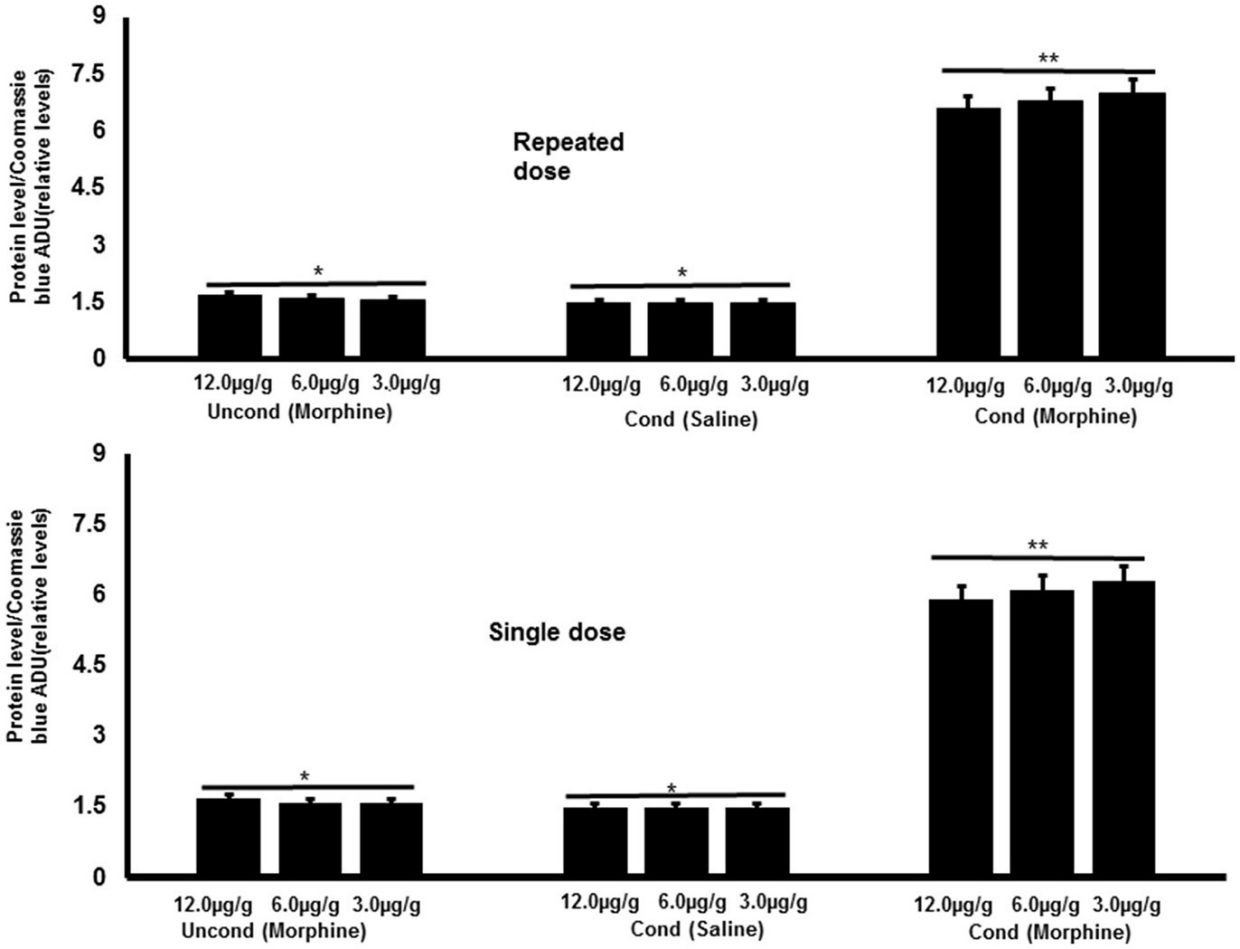
The effect of morphine (3.0, 6.0, and 12.0 μg/g doses) and different environmental treatments on the alteration of c-Fos protein expression for the single and repeated drug treatments regime. *N* = 7 for all treatment doses (3.0, 6.0, and 12.0 μg/g) for each environmental treatment. Normalization was determined with signal intensities of c-Fos proteins to the gels stained with Coomassie blue as a ratio to produce relative abundance units (Dosimetry; ADU). Levels of c-Fos proteins were expressed as a ratio of ADU value to the whole protein in Coomassie blue-stained gels. For the single morphine treatment, there was a significant effect of the environment [*F*_(1, 54)_ = 90.23, *P* < 0.001] such that morphine conditioned environment significantly increased c-Fos (^**^P < 0.05) when compared with the effect of conditioned saline (^*^P < 0.05) or morphine unconditioned environment (^*^P < 0.05). The effect of the environment was also significant for the repeated treatment [*F*_(1, 54)_ = 50.25, *P* < 0.001], such that there was a significant effect of morphine conditioned-environment on the alteration of c-Fos expression (^**^P < 0.05) when compared with saline conditioned (^*^P < 0.05) or morphine unconditioned environment (^*^P < 0.05).

The conditioned association between environmental cues and drug-activated reward circuitry are known to be a key point in drug relapse in humans (Childress et al., 1988; Zahm et al., 2010). The neuronal alterations that occur in this process are linked to certain transcription factors, such as ΔFosB and the cAMP-response component binding protein (CREB), whose activity is altered through changes in gene expression. The c-Fos proteins (catecholamine reuptake transporters) are linked to the morphine response by regulating *Fos* gene expression levels in dopamine neurons (Curran et al., 1996). The c-Fos protein has been studied in mammals in regard to activation of brain regions by drugs of abuse and, when activated, plays a role in signal transduction and genetic modifications. This protein has not been studied extensively in invertebrate models, but an investigation of c-fos gave insights into the molecular alterations associated with drug reward in invertebrates (Dziopa et al., 2011). The single and repeated injections of morphine at 3.0, 6.0, and 12.0 μg/g (Figure 3) in an unconditioned experiment did not reveal a significant increase in c-Fos expression. However, in the conditioned experiment, 5 days of repeated morphine treatments paired with a novel environment produced a significant increase in c-Fos expression. The intensities in c-Fos bands were increased in both single and repeated morphine treatment groups, but were higher in the repeated morphine treatment group. The levels of c-Fos expression remained constant in the control group. This result suggests that novel environment when paired with drugs impacts gene regulatory processes (Dziopa et al., 2011).

**Figure 4:**
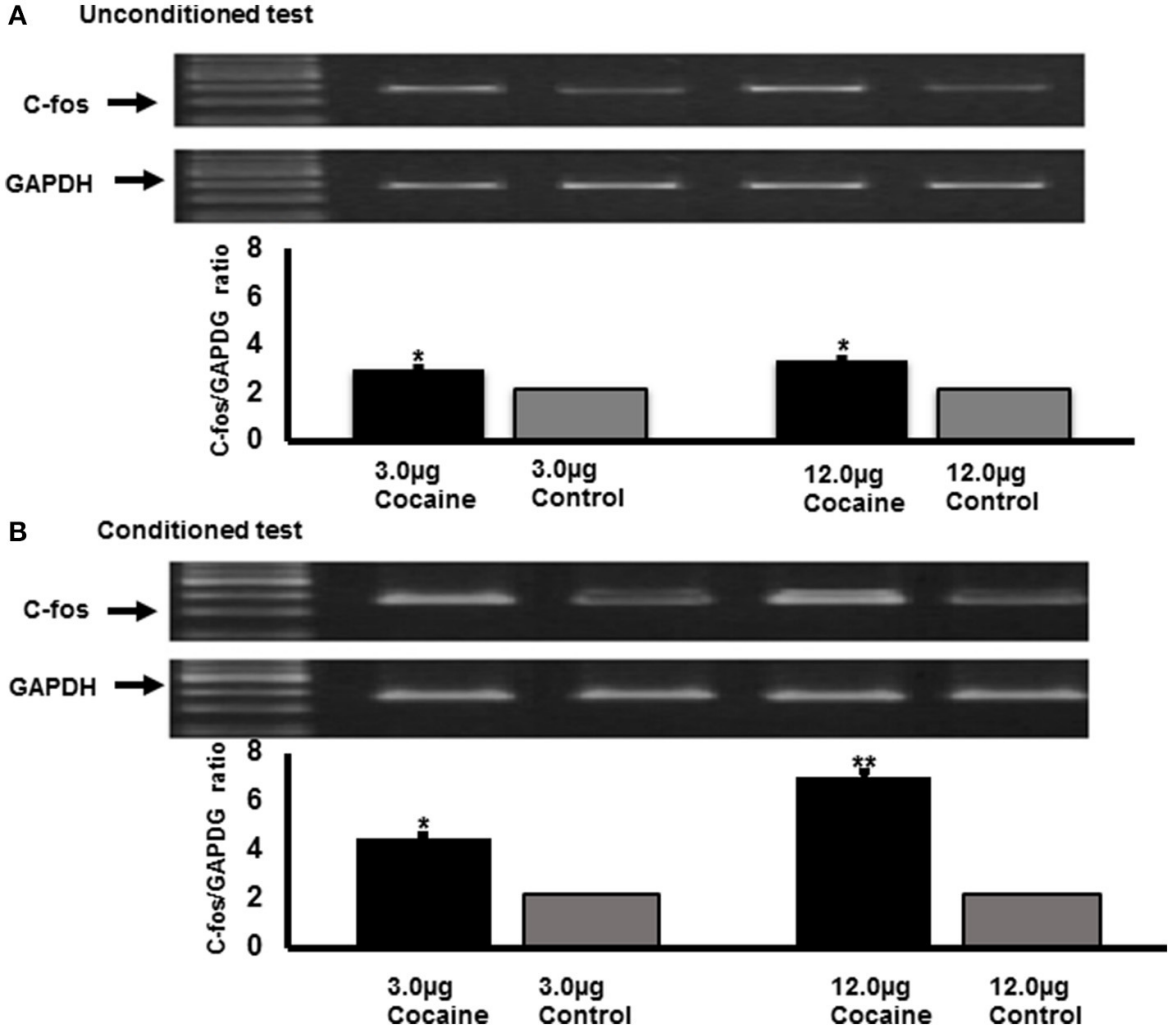
C-Fos mRNA alterations in cocaine treated animals (3 and 12 μg/g) in unconditioned **(A)** and conditioned environment **(B)**. The effect of cocaine on c-Fos mRNA expression was measured by quantitative RT-PCR (Top panel) and normalized with GAPDH (panel below). Data represent mean ± S.E.M. at 35 min following CPP and non-conditioning after 5 days of cocaine injections (*n* = 9). Different doses of cocaine (3 and 12 μg/g) induced a significant [*F*_(3, 23)_ = 62.05, *P* < 0.001] change in c-Fos mRNA expression in unconditioned treatment with cocaine when compared with the control animals without cocaine injection. The effects of 3 and 12 μg/g doses of cocaine were not significantly different (3 μg/g, ^*^P < 0.05; 12 μg/g, ^*^P < 0.05). The conditioning effect of cocaine was significant [*F*_(3, 27)_ = 92.12, *P* < 0.001] when compared with the control group. The expression of C-Fos mRNA was significantly higher at a higher dose of cocaine (12 μg/g, ^**^P < 0.05) when compared with a lower dose (3 μg/g, ^*^P < 0.05).

In a similar study with cocaine (Nathaniel et al., 2012b), there was a significant increase in the expression of c-Fos following the injections of 3.0 and 12.0 μg/g doses of cocaine in a conditioned test when compared with the unconditioned test (Figure 4). Maximal intensities in c-Fos bands were observed with a high dose of cocaine (12.0 μg/g) when compared with a low dose (3.0 μg/g). Collectively, these results show that cocaine-induced reward paired with a hard environment is associated with the enhancement of c-Fos mRNA expression in the accessory lobe of a crayfish (Nathaniel et al., 2012b). This indicates that cocaine produced a context specific reward in the novel hard-texture environment, and that the repeated injections of the drug are also associated with the increase of c-Fos mRNA expression in the accessory lobe of the crayfish. In mammals, c-Fos mRNA markers have been reported as an indication of activated brain regions associated with drug usage, and at specific targets (Zawilska, 2003; Perrotti et al., 2004; Yamada et al., 2007; Zavala et al., 2007; Xu, 2008; Velázquez-Sánchez et al., 2009; Watanabe et al., 2009). The increase of c-Fos mRNA expression in the accessory lobe of the crayfish brain suggests that the accessory lobe of the crayfish may play a role analogous to the higher brain structures in the frontal regions of the cerebral cortex of mammals. Such areas include the medial prefrontal cortex, anterior cingulate cortex, or orbitofrontal cortex, responsible for high-order choices made within its environment in regard to the search for food, shelter or conspecifics (Sandeman et al., 1992).

## Conclusion

These studies offer insight into potential mechanisms that remain unexplored within the crayfish model in drug addiction research. Crayfish as a model organism features a highly modular, experimentally accessible nervous system, yet capable of substantial behavioral complexity. With strongly conserved evolutionary mechanisms for behavioral sensitization, drug dependence, and drug-induced reward seeking, crayfish demonstrate significant vulnerability to human drugs of addiction. Research in crustaceans thus offers a valuable perspective for studying the neural implementation of conserved behavioral phenomena, including motivation, escape, aggression, and drug-sensitive reward.

## Author contributions

AS, VO, and LW, reviewed articles related to this manuscript and wrote the initial draft. AI-N, RH, and TN reviewed the contents, data and the final draft of the manuscript.

### Conflict of interest statement

The authors declare that the research was conducted in the absence of any commercial or financial relationships that could be construed as a potential conflict of interest.
